# Sitagliptin Mitigates Total Body Irradiation-Induced Hematopoietic Injury in Mice

**DOI:** 10.1155/2020/8308616

**Published:** 2020-07-25

**Authors:** Meifang Wang, Yinping Dong, Jing Wu, Hongyan Li, Junling Zhang, Lu Lu, Yuanyang Zhang, Zewei Zhou, Saijun Fan, Deguan Li, Aimin Meng

**Affiliations:** ^1^Tianjin Key Laboratory of Radiation Medicine and Molecular Nuclear Medicine, Institute of Radiation Medicine, Chinese Academy of Medical Sciences & Peking Union Medical College, Tianjin 300192, China; ^2^NHC Key Laboratory of Human Disease Comparative Medicine (The Institute of Laboratory Animal Science, CAMS&PUMC); Beijing Key Laboratory for Animal Models of Emerging and Remerging Infectious Diseases; Beijing Engineering Research Center for Laboratory Animal Models of Human Critical Diseases, Beijing 100021, China

## Abstract

Sitagliptin, an inhibitor of the dipeptidyl peptidase IV (DPP4), has been implicated in the regulation of type 2 diabetes. However, the role and mechanism of sitagliptin administration in total body irradiation (TBI)- induced hematopoietic cells injury are unclear. In this study, we demonstrated that sitagliptin had therapeutic effects on hematopoietic damage, which protected mice from 7.5 Gy TBI-induced death, increased the numbers and colony formation ability of hematopoietic cells. These therapeutic effects might be attributed to the inhibition of NOX4-mediated oxidative stress in hematopoietic cells, and the alleviation of inflammation was also helpful. Therefore, sitagliptin has potential as an effective radiotherapeutic agent for ameliorating TBI-induced hematopoietic injury.

## 1. Introduction

Ionizing radiation (IR) has been widely used in industry, agriculture, and medical therapy, such as nuclear power generation, agricultural breeding, cancer treatment, and so on [1, 2]. However, the risks of accidental nuclear accidents, radiotherapy sequelae, and even nuclear war and nuclear terrorism are gradually rising, which makes the demand for radiation protection and treatment increasing. Exposure to a high dose of IR within a relatively short period of time may induce acute radiation syndromes (ARS), including effects experienced in the hematopoietic system, gastrointestinal system and brain [2–4], and hematopoietic radiation injury is the most common ARS.

The hematopoietic system has a hierarchical structure, in which hematopoietic stem cells (HSCs) is located at the top, which can proliferate downwards into multipotential progenitor cells (MPPs) and hematopoietic progenitor cells (HPCs), and further differentiate into mature blood cells [5, 6]. HPCs show high sensitivity to radiation due to their fast proliferation rate. Middle or high doses of IR can deplete MPPs and HPCs and lead to acute myelosuppression. Then, HSCs proliferate and differentiate to supplement MPPs and HPCs, but persistent myelosuppression occurs with HSCs injury [7, 8]. Radiation-induced myelosuppression is one of the important pathological basis of clinical manifestations of ARS, including infection, hemorrhage, and anemia, so recovery of the hematopoietic system plays an important role in the treatment of radiation damage. The hematopoietic growth factors (HGFs) such as granulocyte colony-stimulating factor (G-CSF) filgrastim and pegfilgrastim and the granulocyte-macrophage colony-stimulating factor (GM-CSF) sargramostim have currently been approved by the US Food and Drug Administration to mitigate hematopoietic abnormalities in ARS in order to improve patients survival [9]. However, the application of HGFs not only may lead to fever, pain, vomiting, and so on, but also destroys the self-renewal ability of HSCs, which accelerates the depletion of HSCs and further affects the long-term recovery of hematopoietic system [10–13]. Therefore, studying the mechanism of regulation of the hematopoietic system and exploring strategies to mitigate hematopoietic radiation damage are urgent problems to be solved.

As an oral hypoglycemic agent approved by FDA, sitagliptin increases the activity of glucagon-like peptide-1 (GLP-1) and glucose-dependent insulinotropic polypeptide by highly selective inactivation of DPP4, thereby promoting insulin secretion from *β*-cells and inhibiting glucagon secretion from *α*-cells, so sitagliptin is widely used in the treatment of type 2 diabetes [14–17]. Studies have shown that sitagliptin can suppress oxidative stress in severe acute pancreatitis-associated intestinal inflammation, diabetic cardiomyopathy, chronic cerebral hypoperfusion, heart failure, liver ischemia-reperfusion, and so on [18–22]. Broxmeyer et al. [23] found that radiation increased the activity of DPP4 in bone marrow (BM) cells, and DPP4 knockout or inhibition before IR prevented the hematopoietic radiation injury. Sitagliptin's target DPP4 exists on the surfaces of a variety of cells including HSCs and HPCs, and partially presents in the circulating blood in soluble form [24, 25]. DPP4 is able to combine with chemokines, colony-stimulating factors (CSFs), and interleukins involved in the regulation of hematopoietic system [26], inhibiting its activity is beneficial to homing and implantation of hematopoietic cells [27]. However, the therapeutic effects and the mechanism of sitagliptin in the treatment of hematopoietic radiation damage remain to be studied.

In this article, we investigated the therapeutic role of sitagliptin in hematopoietic radiation injury and its underlying mechanisms. Our results demonstrated that the administration of sitagliptin had therapeutic effects on TBI-induced hematopoietic damage, which protected mice from TBI-induced death, increased the numbers of hematopoietic cells and the proliferation ability of HPCs. In addition, sitagliptin not only inhibited NOX4-mediated oxidative stress response in hematopoietic cells, but also might mitigate inflammation.

## 2. Materials and Methods

### 2.1. Reagents

Biotin conjugated anti-mouse-CD4 (clone 34 GK1.5), anti-mouse-CD8 (clone 53-6.7), anti-mouse-CD11b (clone M1/70), anti-mouse-CD45R/B220 (clone RA3-6B2), anti-mouse-Ly6G/Gr-1 (clone RB68C5), anti-mouse-Ter119 (clone Ter119), anti-mouse-CD117 (c-kit)-APC (clone 2B8), anti-mouse -Ly-6A/EA (Sca-1)-PE (clone D7), and PERCP-conjugated streptavidin were purchased from eBioscience (San Diego, CA, USA). In addition, 2,7-dichlorodihydrofluorescein diacetate (DCFDA) was purchased from Sigma (St. Louis, MO, USA). MethoCult GF M3534 medium was purchased from Stem Cell Technologies (Vancouver, Canada). MitSox red mitochondrial superoxide indicator was obtained from Life Technologies (Grand Island, NY, USA). Rabbit anti-*γ*H2AX was obtained from Cell Signaling Technology (Danvers, MA, USA). Rabbit anti-NOX4 was obtained from Proteintech (Wuhan, China). FITC-conjugated goat anti-rabbit antibodies were obtained from Abcam Biotechnology (Cambridge, MA, USA). Cytofix/Cytoperm buffer (554722), Perm/Wash buffer (554723), and Cytoperm Permeabilization Buffer Plus (561651) were obtained from BD Pharmingen (San Diego, CA, USA). Sitagliptin was obtained from Merck Sharp & Dohme (South Granville, NSW, Australia).

### 2.2. Animals

Male C57BL/6J mice weighing 20-22 g were purchased from Beijing HFK Bioscience Co, Ltd. (Beijing, China) and housed in the certified animal facility at the Institute of Radiation Medicine of the Chinese Academy of Medical Sciences (CAMS). All mice were randomly divided into different groups one week prior to the study to allow for acclimatization. All procedures involving animal experiments were conducted in accordance with a protocol approved by the Institutional Animal Care and Use Committee of CAMS.

### 2.3. Irradiation and Treatment

Mice were randomly assigned to 4 groups: control, sitagliptin, TBI, and TBI+sitagliptin in survival experiment and assigned to 3 groups: control, TBI, and TBI+sitagliptin in other experiments. Mice were exposed to a LD50 dose (7.5 Gy) TBI for the survival study or sublethal dose (4 Gy) TBI for experiments using a ^137^Cs source housed in an Exposure Instrument Gammacell-40 (Atomic Energy of Canada Lim) at a dose rate of 1.0 Gy per min. For sitagliptin treatment, mice were treated with 10 mg/kg sitagliptin via oral administration once daily for 7 d; the first dose was administered 2 hours after TBI. The determination of the dose for mice was based on the conversion of the recommended dose for humans (100 mg/kg). Mice in the control and TBI groups were given PBS in the same protocol. In the 7.5 Gy irradiation experiment, 10 mice were used in each group, while in the 4 Gy irradiation experiment, 5 mice per group. 10 days after 4 Gy TBI, the mice were sacrificed and samples were collected [13].

### 2.4. Analysis of the Numbers of Bone Marrow Mononuclear Cells (BMMNCs), HPCs, and HSCs

BM cells were flushed from mouse femurs with PBS, and the numbers of BMMNCs were counted using a MEK-7222k hemocytometer (NIHON KOHDEN, Tokyo, Japan) and expressed as ×10^6^/femur. BM cells were incubated with biotin-conjugated lineage antibodies specific for murine CD4, CD8, Ter119, CD11b, CD45R/B220, and Gr-1, and stained with streptavidin-PerCp, Sca1-PE, and c-kit-APC. The numbers of HPCs (lin^−^ c-kit^+^ Sca-1^−^) and HSCs (lin^−^ c-kit^+^ Sca-1^+^, LSK) were calculated using the following equation: percentage × BMMNCs/femur [13].

### 2.5. Colony Forming Unit-Granulocyte Macrophages (CFU-GM) Assay

The CFU-GM assays were conducted by culturing BM cells in MethoCult GF M3534 methylcellulose medium (Stemcell Technologies, Vancouver, BC). The colonies of CFU-GM were counted on day 7 according to the manufacturer's protocol. The results were presented as the numbers of CFU-GMs per 2 × 10^4^ cells [28].

### 2.6. Competitive Repopulation Assay (CRA)

In the present study, donor cells (1 × 10^6^ BMMNCs) were collected from C57BL/6-Ly-5.1 (CD45.1) mice after they received various treatments and mixed with 1 × 10^6^ competitive BMMNCs from C57BL/6J (CD45.2) mice. The mixed cells were transplanted into lethally irradiated (9.0 Gy TBI) C57BL/6J (CD45.2) recipient mice through lateral canthus vein injection. The percentage of donor-derived (CD45.1 positive) cells in the recipients' peripheral blood was examined 2 months after transplantation. The red blood cells (RBCs) were lysed using RBC lysis solution (eBioscience), and then the blood samples were stained with the following antibodies: anti-CD45.1-FITC, anti-CD45.2-PE. The cells were analyzed with an Accuri C6 flow cytometer (BD Bioscience) [28, 29].

### 2.7. Analysis of the Levels of Intracellular Reactive Oxygen Species (ROS)

After the BM cells were stained with the LSK antibodies as described above, the cells were incubated with 10 *μ*M DCFDA or 5 *μ*M MitSox for 20 min at 37°C. The intracellular ROS levels in hematopoietic cells were analyzed by measuring the mean fluorescence intensity (MFI) of DCF and MitSox by flow cytometry. For each sample, a minimum of 100,000 Lin^−^ cells were acquired [30].

### 2.8. Analysis of *γ*H2AX Phosphorylation and NOX4 Expression

After the BM cells were stained with the LSK antibodies as described above, the cells were fixed and permeabilized by BD Cytofix/Cytoperm buffer according to the manufacturer's protocol and then stained with antibodies against *γ*H2AX phosphorylation or NOX4 and FITC-conjugated secondary antibodies. The expression of *γ*H2AX and NOX4 in the hematopoietic cells was determined by analyzing the MFI of FITC by flow cytometry [30].

### 2.9. Measurement of Inflammatory Cytokines in Serum

10 days after irradiation, the peripheral blood of mice was collected, and the serum was taken after standing overnight. Then, the serum was analyzed using the BD Cytometric Bead Array Mouse Inflammation Kit (San Diego, CA, USA) as the manufactures' protocol. In brief, the samples were incubated with mixed capture beads and detection antibodies. After incubation for two hours at room temperature, the samples were washed and detected by flow cytometry. The results were analyzed by the company.

### 2.10. Statistical Analysis

Data were presented as the mean ± standard error of the mean. Significant differences between experimental groups were evaluated by using a one-way analysis of variance (ANOVA) with repeated measures followed by post hoc comparisons with Tukey's multiple paired comparison test except result 2. Significant differences between groups of the numbers of hematopoietic cells were evaluated by unpaired two-tailed Student's *t* test. Mice survival curves were analyzed by the Kaplan-Meier method and log-rank tests. Differences were considered significant at *p* < 0.05. Statistical analyses were performed using GraphPad Prism 8 software (SanDiego, CA, USA).

## 3. Results

### 3.1. Sitagliptin Increased the Survival Rate of Mice after TBI

**Figure 1:**
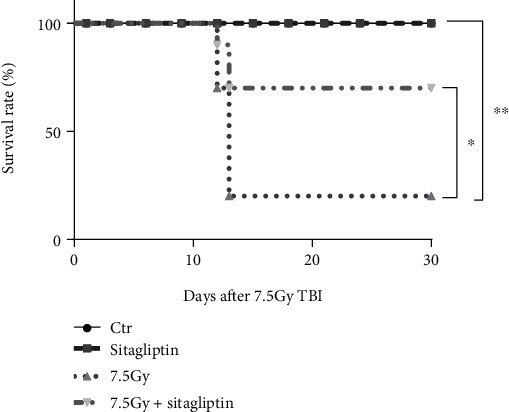
Effects of sitagliptin on the survival of mice exposed to 7.5 Gy TBI. Mice were divided into 4 groups: the control group and 7.5 Gy group were intragastrically administrated with PBS, and the sitagliptin group and 7.5 Gy+sitagliptin group were intragastrically administrated with sitagliptin. The drugs were given for the first time 2 hours after the 7.5 Gy TBI, followed by continuous administration for 7 days, and the survival of the mice was observed for 30 days. Kaplan-Meier survival analysis of mice after TBI, *n* = 10, ^∗^*p* < 0.05, ^∗∗^*p* < 0.01.

In order to test whether sitagliptin affected the survival of mice after TBI, we treated mice with 10 mg/kg sitagliptin daily for 7 days after 7.5 Gy TBI and observed their 30-day survival rate. As shown in Figure 1, the Kaplan-Meier analysis of survival indicated that the survival rate of irradiated mice treated with sitagliptin was significantly higher than that of 7.5 Gy irradiated mice.

### 3.2. Sitagliptin Increased the Numbers of Hematopoietic Cells after TBI

**Figure 2:**
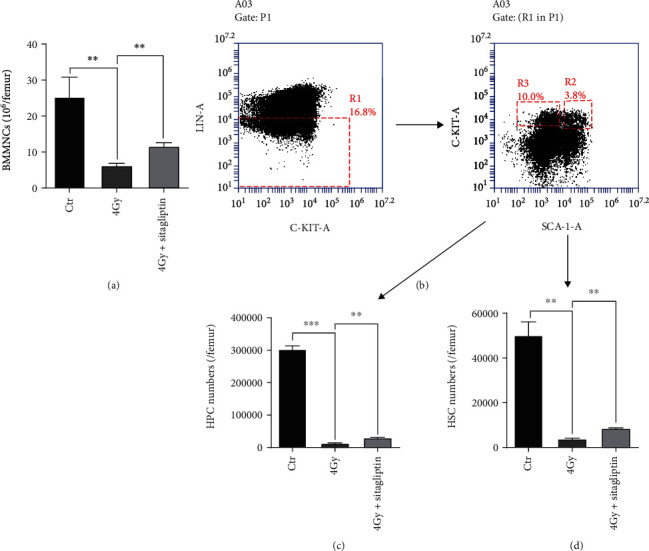
Effects of sitagliptin on the numbers of hematopoietic cells. Mice were divided into 3 groups: sham irradiation, 4 Gy group, and 4 Gy+sitagliptin group. The dosage regimen was the same as above. BM cells were collected from mice 10 days after TBI. (a) Numbers of BMMNCs; (b) Representative flow cytometry gate graph of lineage negative and HSPCs; (c) Numbers of HPCs; (d) Numbers of HSCs. Data were expressed as the mean ± SEM (*n* = 5). ^∗∗^*p* < 0.01, ^∗∗∗^*p* < 0.001.

The survival of mice exposed to sublethal dose radiation can partly attribute to the recovery of the hematopoietic system [13, 28]. In the present study, the numbers of BMMNCs and HSPCs in BM were also analyzed. 4 Gy TBI caused a decrease in the numbers of BMMNCs and HSPCs compared with that from control mice. However, sitagliptin mitigated the impaired BMMNCs and HSPCs in BM (Figure 2). These data suggested that sitagliptin effectively relieved 4 Gy TBI-induced hematopoietic cell injury.

### 3.3. Sitagliptin Influenced the Functions of HSPCs after TBI

**Figure 3:**
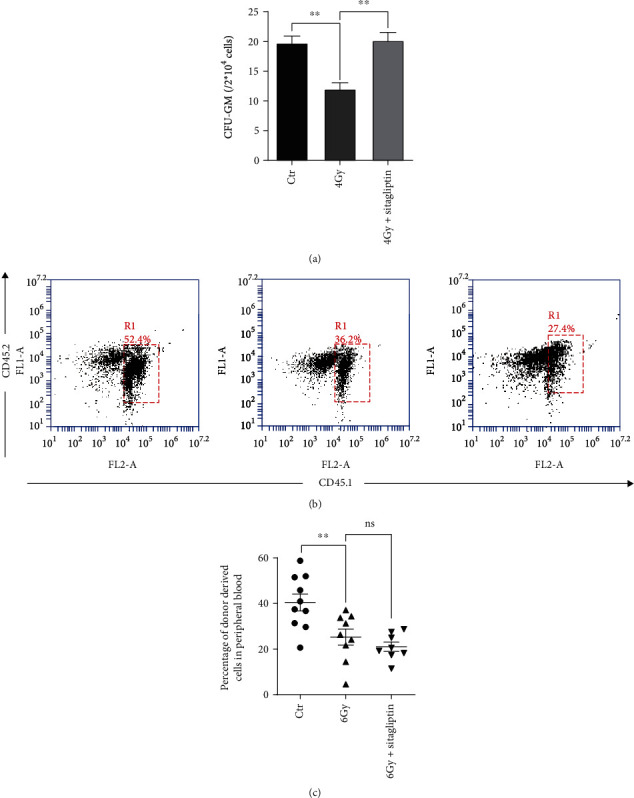
Effects of sitagliptin on the functions of HSPCs. The grouping and administration methods are the same as above. BM cells were collected from mice 10 days after TBI. (a) BM cells were cultured in MethoCult GF M3534 methyl ligand medium, and the numbers of CFU-GMs were counted after 7 days. The proportion of donor cells in the recipient mice was measured 2 months after the donor cells were transplanted to the recipient mice; (b) Representative FACS analysis of the CRA; (c) The percentage of donor-derived cells in peripheral blood cells. Data were expressed as the mean ± SEM (*n* = 5), ^∗∗^*p* < 0.01.

BM exposed to moderate or high-dose TBI may have long-term hematopoietic residual damage, mainly due to defects in the self-renewal and differentiation ability of HSCs [7]. Thus, we analyzed the effects of sitagliptin on the clonogenic function of HPCs in mice exposed to 4 Gy via CFU assays and the engraftment capability via CRA. As shown in Figure 3(a), 4 Gy TBI caused a significant suppression of HPCs clonogenic function, and sitagliptin increased the formation of CFU-GMs. Since long-term and repeated transplantation are the gold standard for measuring HSCs functions [31], we performed a CRA to determine whether sitagliptin improved HSC self-renewal function. Our results showed that the engraftment capability of irradiated HSCs did not improve after sitagliptin treatment (Figures 3(b) and 3(c)). These results suggested that sitagliptin had no obvious protective effect on the self-renewal of HSCs.

### 3.4. Sitagliptin Reduced TBI-Induced DNA Double-Strand Breaks (DSBs)

**Figure 4:**
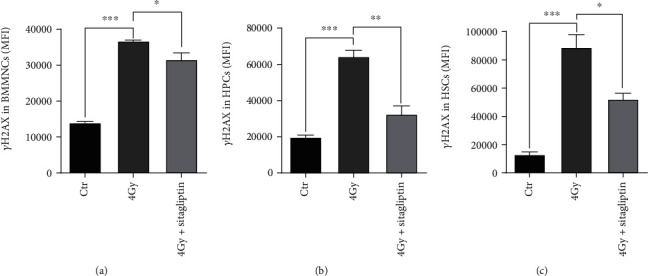
Effects of sitagliptin on the IR induced DNA injury of the hematopoietic cells. Grouping and administration methods as described above. Fixed and permeabilized BM cells after LSK antibodies incubation, then stained with *γ*H2AX phosphorylation antibody. (a) *γ*H2AX formation in BMMNCs; (b) *γ*H2AX formation in HPCs; (c) *γ*H2AX formation in HSCs. Data were expressed as the mean ± SEM (*n* = 5), ^∗^*p* < 0.05, ^∗∗^*p* < 0.01, ^∗∗∗^*p* < 0.001.

As reported previously, TBI caused sustained DNA damage and oxidative DNA damage [32]. To evaluate whether sitagliptin regulated DNA damage of hematopoietic cells, we used flow cytometry to analyze histone H2AX phosphorylation. As shown in Figure 4, compared with the control group, the expression of histone H2AX phosphorylation was higher in BMMNCs, HPCs, and HSCs when the mice exposed 4 Gy TBI, consistent with our previous finding [13, 33]. These data suggested that sitagliptin effectively decreased TBI-induced persistent DNA damage.

### 3.5. Sitagliptin Decreased TBI-Induced Oxidative Stress Levels in Hematopoietic Cells

**Figure 5:**
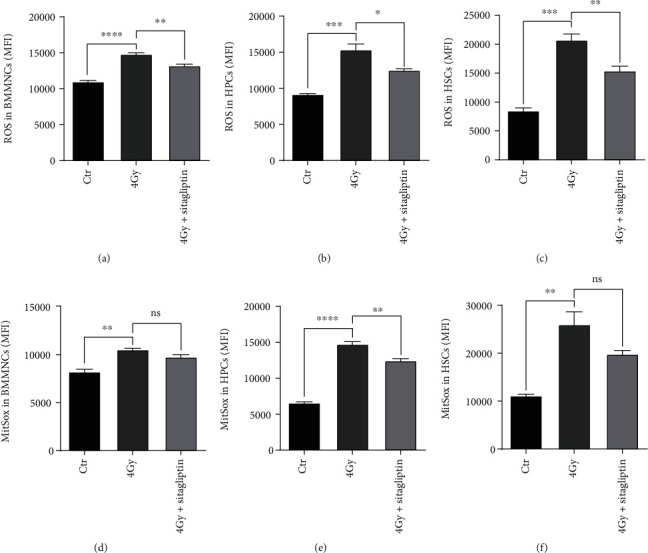
Effects of sitagliptin on the oxidative stress levels of hematopoietic cells. Grouping and administration methods as described above. After 10 days of TBI, BM cells were collected and labeled with LSK antibodies, then incubated with DCFDA or MitSox. (a) ROS of BMMNCs; (b) ROS of HPCs; (c) ROS of HSCs; (d) MitSox of BMMNCs; (e) MitSox of HPCs; (f) MitSox of HSCs. Data were expressed as the mean ± SEM (*n* = 3), ^∗^*p* < 0.05, ^∗∗^*p* < 0.01, ^∗∗∗^*p* < 0.001, ^∗∗∗∗^*p* < 0.0001.

In our previous studies, we have demonstrated that the mice exposed to sublethal doses develop long-term myelosuppression through chronic oxidative stress [34, 35], thus we examined whether sitagliptin ameliorated TBI-induced BM suppression via decreasing ROS levels. In our study, we detected ROS and mitochondrial superoxide radicals by using DCFH-DA and MitSox, respectively. As shown in Figures 5(a)–5(c), compared with those in the control mice, the levels of ROS in mice receiving 4 Gy TBI elevated significantly. When treated with sitagliptin, the ROS levels in BMMNCs and HSPCs decreased obviously. In addition, sitagliptin also decreased the levels of MitSox in hematopoietic cells especially in HPCs (Figures 5(d)–5(f)). These results indicated that sitagliptin decreased oxidative stress in hematopoietic cells.

### 3.6. Sitagliptin Reduced the Expression of NOX4 after TBI

**Figure 6:**
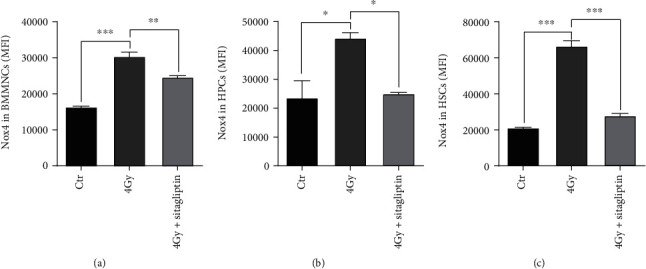
Effects of sitagliptin on the NOX4 expression of hematopoietic cells. Grouping and administration methods as described above. Fixed and permeabilized BM cells after LSK antibodies incubation as mentioned above, then, stained with NOX4 antibody. (a) NOX4 expression in BMMNCs; (b) NOX4 expression in HPCs; (c) NOX4 expression in HSCs. Data were expressed as the mean ± SEM (*n* = 3), ^∗^*p* < 0.05, ^∗∗^*p* < 0.01, ^∗∗∗^*p* < 0.001.

NOX4 is a prooxidase that has been shown to mediate IR-induced increases in ROS production in HSCs [28]. Therefore, we examined the effects of DPP4's inhibition on the expression of NOX4. As shown in Figure 6, an increase in NOX4 expression was detected in BMMNCs, HPCs, and HSCs in the irradiation group compared with the control group, respectively. Sitagliptin decreased the expression of NOX4 in hematopoietic cells. These findings suggested that sitagliptin decreased the levels of ROS in hematopoietic cells in part via a downregulation of NOX4 expression.

### 3.7. Sitagliptin Relieved TBI-Induced Inflammatory Response

**Figure 7:**
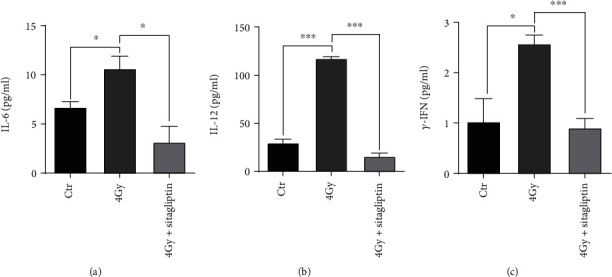
Effects of sitagliptin on the expression of cytokines in the serum. The peripheral blood of mice was collected 10 days after TBI, then left to stand overnight to separate serum and detected by an inflammatory factor kit. (a) IL-6 cytokine; (b) IL-12 cytokine; (c) *γ*-IFN cytokine. Data were expressed as the mean ± SEM (*n* = 3 − 5), ^∗^*p* < 0.05, ^∗∗∗^*p* < 0.001.

DPP4 cleaves the N-terminus of GM-CSF, G-CSF, IL-3, and erythropoietin, and the inhibition of DPP4 enhances their activity [23], so we examined the effect of sitagliptin on the expression of inflammatory cytokines in serum. In our study, we found that sublethal dose irradiation increased the expression of IL-6, IL-12, and *γ*-IFN in mice, while sitagliptin significantly reduced the expression of cytokines (Figure 7). These results suggested that sitagliptin might influence the level of inflammation in the BM microenvironment.

## 4. Discussion

Sitagliptin is a type 2 diabetes treatment drug, which acts by inhibiting the activity of DPP4. In recent years, sitagliptin was found to have antioxidant and anti-inflammatory effects, which plays a role in atherosclerosis, inflammatory bowel disease, heart failure, vascular inflammation, and other diseases [18, 36, 37]. Metformin as another kind of type 2 diabetes drug approved by FDA, our previous study has shown that it alleviates HSCs aging by inhibiting NOX4-mediated oxidative stress, thus improving long-term HSCs injury induced by IR in mice [13]. In addition, metformin improves ARS symptoms such as pulmonary fibrosis and skin collagen deposition [37, 38]. Therefore, we speculate that sitagliptin may also have therapeutic effects on IR-induced tissue damage. In this study, we observed the effect of sitagliptin on the survival rate of irradiated mice and showed that sitagliptin significantly increased the 30-day survival rate, which indicated that sitagliptin had a therapeutic effect on radiation injury in mice.

Then, the therapeutic effects of sitagliptin on hematopoietic radiation injury were explored. Firstly, the changes in the numbers of hematopoietic cells were observed. The results showed that the numbers of BMMNCs, HPCs, and HSCs in mice exposed to IR increased after the administration of sitagliptin, which indicated that sitagliptin could decrease hematopoietic radiation damage. Secondly, the effects of sitagliptin on the function of HPCs and HSCs were evaluated by CFU-GM and CRA experiments. The CFU-GM results showed that sitagliptin could restore the proliferation ability of HPCs, but the CRA results suggested that sitagliptin had no obvious direct effect on the self-renewal of HSCs. It may be due to part of DPP4 is soluble and exists in the microenvironment [25, 39]. Sitagliptin may play a protective role in the hematopoietic injury by direct regulation of hematopoietic cells and indirect action on the hematopoietic microenvironment, which is partly proved by our following serum cytokines results (Figure 7). The oxidative stress induced by IR is an important reason of hematopoietic injury. At the instant of irradiation, IR will cause radiation decomposition of intracellular water and stimulate nitrogen oxide synthase to produce ROS and reactive nitrogen species (RNS), respectively [39]. Radiation also leads to electron leakage of mitochondria, increases expression of cyclooxygenase and lipoxygenase, and changes in NOXs expression [40, 41], resulting in the production of a large numbers of cell-derived free radicals, giving rise to long-term damage to cells. Radiation-induced DNA damage and oxidative stress lead to an increase in the numbers of apoptotic, necrotic, autophagic, and senescent cells [42]. The products of dead cells can trigger inflammation of immune cells and activate the expression of TGF-*β* [43], which in turn lead to the upregulation of NOXs expression; NOXs further amplifies reactions such as oxidative stress in the positive feedback loop and aggravates radiation damage. There are several isoforms of NOXs in nonphagocytic cells, including NOX1, NOX2, NOX3, NOX4, NOX5, DUOX1, and DUOX2 [44]. Previous studies have shown that NOXs, especially NOX4, might be the main reason for TBI-induced ROS production in HSCs [45]. In our previous studies, we have demonstrated that many compounds such as metformin, resveratrol, and 3,3′-diindolylmethane protect hematopoietic radiation injury by inhibiting NOX4 [13, 35, 45]. Recent studies also showed that melatonin alleviated the injury of the radiation-induced hematopoietic system by inhibiting the expression of NOX2 and NOX4 [46]. In this study, we observed that sitagliptin significantly decreased the oxidation level in BMMNCs, HPCs, and HSCs by inhibiting the expression of NOX4. Therefore, NOX4 is a promising target for the treatment of IR-induced hematopoietic injury, and targeting the promotion or inhibition of this enzyme family may mitigate radiation damage to certain organs, such as hematopoietic system, gastrointestinal system, central nervous system, and skin system.

IR not only induces DNA structural damage directly through the ionizing photons, but also destroys DNA structure caused by the increase of ROS [47]. The destruction of DNA structure will lead to metabolic and functional changes and eventually lead to cell damage or death. In this study, we observed the relationship between sitagliptin and DNA damage and found that sitagliptin decreased the expression of *γ*H2AX, consistent with the research in chronic cerebral hypoperfusion mice [48]. These results indicated that sitagliptin alleviated cellular DNA damage and exerted hematopoietic radiation therapy.

Medium or high doses of IR not only damages the hematopoietic system, but also causes injury to the gastrointestinal tract, resulting in intestinal microorganisms to enter the systemic circulation through penetrating mucous membrane [49]. Endotoxins in bacteria will directly interact with cells including endothelial cells in the bone marrow microenvironment, changing the release ability of inflammatory factors, thus inducing myelosuppression and HSCs failure [50]. Studies have shown that rBPI21 reduces the injury and death of HSCs by reducing the level of inflammation and promoting the expression of CSFs in plasma and bone marrow [51]. Sitagliptin exerts a comprehensive and effective anti-inflammatory action on humans, which reduces the concentrations of CRP and IL-6 in plasma [17]. In addition, previous studies have shown that DPP4 may be involved in the expression of IL-6 and other cytokines in the JAK-STAT signaling pathway, while the JAK-STAT signaling pathway is involved in the differentiation, activation, and proliferation of Th cells, and the expression of Th1 cytokines (*γ*-IFN, IL-2, TNF-*α*, etc.) or Th2 cytokines (IL-4, IL-6, IL-10, etc.) is decreased after blocking the JAK-STAT pathway; therefore, the inflammatory response is alleviated [52, 53]. In our study, we observed the relationship between sitagliptin and the expression of inflammatory cytokines IL-6, IL-12, and *γ*-IFN in serum. It was found that sublethal dose irradiation increased the expression of IL-6, IL-12, and *γ*-IFN in mice, while sitagliptin significantly reduced the expression of cytokines (Figure 7). These suggested that sitagliptin might treat hematopoietic injury from IR by influencing cytokines in the BM microenvironment.

## 5. Conclusions

In conclusion, our study showed that the administration of sitagliptin had therapeutic effects on hematopoietic injury. The therapeutic effect might be mainly achieved by reducing the level of NOX4-mediated oxidative stress in hematopoietic cells, and the alleviation of inflammatory was also helpful. Therefore, sitagliptin might be a potential therapeutic agent for the treatment of radiation-induced hematopoietic injury.

## Data Availability

The data used to support the findings of this study are available from the corresponding author upon request.
